# P**-**cresol and Indoxyl Sulfate Impair Osteogenic Differentiation by Triggering Mesenchymal Stem Cell Senescence

**DOI:** 10.7150/ijms.48492

**Published:** 2021-01-01

**Authors:** Witchayapon Kamprom, Tulyapruek Tawonsawatruk, Sumana Mas-Oodi, Korrarit Anansilp, Manoch Rattanasompattikul, Aungkura Supokawej

**Affiliations:** 1 Department of Clinical Microbiology and Applied Technology, Faculty of Medical Technology, Mahidol University, Salaya, Nakhon Pathom, Thailand; 2 Department of Orthopaedics, Faculty of Medicine Ramathibodi Hospital, Mahidol University, Bangkok, Thailand; 3 International Center for Medical and Radiological Technology, Faculty of Medical Technology, Mahidol University, Salaya, Nakhon Pathom, Thailand; 4 Medical Department, Golden Jubilee Medical Center, Faculty of Medicine Siriraj Hospital; 5 Department of Clinical Microscopy, Faculty of Medical Technology, Mahidol University, Salaya, Nakhon Pathom, Thailand

**Keywords:** p-cresol, indoxyl sulfate, chronic kidney disease, osteogenesis, cellular senescence, mesenchymal stem cells

## Abstract

Chronic kidney disease (CKD) patients obtained high levels of uremic toxins progressively develop several complications including bone fractures. Protein-bound uremic toxins especially p-cresol and indoxyl sulfate are hardly eliminated due to their high molecular weight. Thus, the abnormality of bone in CKD patient could be potentially resulted from the accumulation of uremic toxins. To determine whether protein-bound uremic toxins have an impact on osteogenesis, mesenchymal stem cells were treated with either p-cresol or indoxyl sulfate under *in vitro* osteogenic differentiation. The effects of uremic toxins on MSC-osteoblastic differentiation were investigated by evaluation of bone phenotype. The results demonstrated that p-cresol and indoxyl sulfate down-regulated the transcriptional level of collagen type I, deceased alkaline phosphatase activity, and impaired mineralization of MSC-osteoblastic cells. Furthermore, p-cresol and indoxyl sulfate gradually increased senescence-associated beta-galactosidase positive cells while upregulated the expression of *p21* which participate in senescent process. Our findings clearly revealed that the presence of uremic toxins dose-dependently influenced a gradual deterioration of osteogenesis. The effects partially mediate through the activation of senescence-associated gene lead to the impairment of osteogenesis. Therefore, the management of cellular senescence triggered by uremic toxins could be considered as an alternative therapeutic approach to prevent bone abnormality in CKD patients.

## Introduction

Chronic kidney disease **(**CKD**)** is globally known as the major health issues influenced the leading cause of death worldwide **1.** The pathological effect of CKD is mainly from the accumulation of waste products due to the reduction of the renal clearance activity**.** The retention of various solutes in blood circulation of CKD patients has been considered as the factors contributing to the pathological effect of CKD and its complications**2.** Uremic toxins are the uremic solute comprised of three types including free**-**water soluble low molecular weight molecules, middle molecules, and protein**-**bound uremic toxins **3.** These uremic toxins are normally secreted by the urinary system**.** CKD patient with stage 3**-**5 are diagnosed with the reduction of glomerular filtration rate **(**GFR**).** Therefore, the level of uremic toxins turned to be high, although those patients were treated by renal dialysis but some high molecular weight uremic toxins cannot be removed by the dialysis membrane**.** The impacts of uremic toxin to the health status of CKD patients were determined, for example, cardiovascular disease **4**, anemia **5, 6** as well as bone abnormality**7.** However, the underlying mechanism was largely unknown**.** The previous studies demonstrated that indoxyl sulfate increased reactive oxygen species **(**ROS**)** production and promote oxidative stress in endothelial cell**8** and vascular smooth muscle cell **9, 10** resulting in cellular senescence**.** Those impacts of indoxyl sulfate may at least mediate the development of cardiovascular disease**10** and promote the progression of CKD**.** For anemia, previous study demonstrated that indoxyl sulfate could suppress EPO production by inhibiting the activation of HIF **11.** The involvement of indoxyl sulfate in cellular senescence induction was shown by increasing the expression of p53 and p21 in proximal tubular cells **12** and endothelial cells **13.** In addition, indoxyl sulfate affected the expression of klotho; an anti**-**aging gene which highly expressed in kidney cell **14.** Thus, several complications occurred in CKD patient potentially result from the effect of uremic toxins in induction of cellular senescence**.**

The increased risk of fracture in CKD patients originate from the alteration of bone metabolism and formation**7.** This effect associated with the disturbance of mineral metabolism including calcium, phosphate, parathyroid hormone **(**PTH**)**, or vitamin D resulting in bone abnormalities both in quality and quantity**15.** Nonetheless, the effect of uremic toxins was not clearly identified**.** Among uremic toxin, p**-**cresol and indoxyl sulfate are the prominent protein**-**bound uremic toxins presented at high levels especially in the end**-**stage renal diseases **(**ESRD**) 16, 17.** P**-**cresol and indoxyl sulfate are accumulated due to their strong protein binding properties which unable to eliminate through the membrane dialysis**18.** These remarkable uremic toxins effect the biological function of tissue via promotion of inflammation and oxidative stress**19.** Moreover, recent studies suggested that indoxyl sulfate could inhibit osteoclast differentiation **20** and impaired PTH signaling which is an important signal for regulation of bone metabolism**21.**

Taken together, p**-**cresol and indoxyl sulfate may be an important factor that interfere bone formation and bone resorption**.** Bone abnormality may be mediated at least in part by cellular senescence induced under uremic toxin conditions**.** Although, the cytopathic effects of indoxyl sulfate on osteoblastic cell line have been investigated **22**, the roles of p**-**cresol and indoxyl sulfate on osteogenesis of mesenchymal stem cells** (**MSCs**)** have not yet been clarified**.** It is necessary to better understand the effect of p**-**cresol and indoxyl sulfate on each stage of osteogenesis and osteoblast functions**.** Thus, we aim to determine osteogenic differentiation ability of MSCs in the presence of a certain protein**-**bound uremic toxin**.** MSCs are capable of differentiation toward fat cells, cartilage cells, and bone cells**.** Nowadays, MSCs were applied as a promising cell source for studying the disease modeling, particularly in musculoskeletal problems**.** The *in vitro* osteogenesis was performed by culture MSCs with the osteogenic induction medium followed by evaluation of bone phenotype **23.** The series of osteoblastic differentiation in MSCs were assessed in the presence or absence of various concentrations of p**-**cresol and indoxyl sulfate by measuring alkaline phosphatase activity, mineralization, and osteogenic gene expression**.** The cellular senescence and its associated gene expression were also determined to encompass pathogenesis of CKD with bone defect**.** Understanding the role of protein**-**bound uremic toxins not only lead to prevention of the disease progression but also promote the development of new therapeutic approach to treat bone disease in CKD patients in the future**.**

## Materials and Methods

### Subjects

This study was approved by the Ramathibodi Institutional Review Board, Faculty of Medicine Ramathibodi Hospital, Mahidol University in accordance with the Declaration of Helsinki **(**protocol number ID 08**-**60**-**63**).** All human bone marrow samples were obtained from healthy volunteers after written informed consent**.**

### Bone marrow*-*derived mesenchymal stem cells isolation and culture

Bone marrow**-**derived mesenchymal stem cells **(**MSCs**)** were isolated from mononuclear cells, prepared by Histopaque1**.**077 **(**Merck Millipore, USA**)** with density gradient centrifugation as previously described**24.** Bone marrow mononuclear cells were re**-**suspended with complete medium that consisted of low**-**glucose Dulbecco**'**s Modified Eagle**'**s medium **(**DMEM**-**LG; GIBCO, USA**)**, 10**% (**v**/**v**)** Fetal Bovine Serum **(**FBS; Merck Millipore, USA**)**, GlutaMAX **(**GIBCO^TM^, Invitrogen Corporation, USA**)**, and penicillin**-**streptomycin **(**GIBCO, Invitrogen Corporation, USA**).** The cells were maintained at 37˚C in humidified atmosphere containing 5**%** CO_2_**.** After 48**-**hour incubation, non**-**adherent cells were removed**.** The medium was changed twice a week until reaching 80**%** of confluent**.**

MSCs were investigated for the expression of MSC surface markers in according to minimal criteria of MSCs stated by ISCT**25**, including CD73, CD90, CD105** (**positive markers**)** and hematopoietic markers **(**negative markers**)** including CD34, CD45**.** The capacities to differentiate toward osteoblast and adipocyte were determined by cultivation MSCs in osteogenic**-** and adipogenic**-**induction medium**.** Osteogenic and adipogenic phenotype were evaluated at day 14**.** Cells were stained with alizarin red S for detection of calcium deposition in osteoblast and stained with oil red O for detection of adipogenic cells**.** Only the characterized MSCs at 3^rd^**-**6^th^ passage were included in this experimental studies**.**

### Effect of indoxyl sulfate and p*-*cresol on MSCs viability

1x10^4^ MSCs were cultured in complete medium with or without several concentrations of p**-**cresol **(**10, 40, 80, and 160 µg**/**ml which equal to 94**.**22, 376**.**86, 753**.**72, and 1507**.**44 µM, respectively; Sigma**-**Aldrich, USA**)** or indoxyl sulfate **(**25, 50, 100, and 200 µg**/**ml which equal to 99**.**48, 198**.**97, 397**.**93, and 795**.**86 µM, respectively; Sigma**-**Aldrich, USA**).** The morphology of MSCs was observed under inverted microscope **(**Olympus, Japan**).** The effects of indoxyl sulfate and p**-**cresol on MSCs were investigated after treatment for 24, 48, and 72 hours using MTT assay**.** The absorbance of soluble formazan crystals of each sample was inspected**.** The cell viability of individual conditions was calculated as percent viable cells of control**.**

### The effect of indoxyl sulfate and p*-*cresol on osteogenic differentiation of MSCs

To investigate the impact of p**-**cresol and indoxyl sulfate to *in vitro* osteogenesis of MSCs, 1x10^5^ MSCs were cultured in osteogenic induction medium consisting of DMEM**-**LG, 10**% (**v**/**v**)** FBS, penicillin**-**streptomycin, 10 mM β**-**glycerophosphate, 0**.**1 µM dexamethasone, and 50 µg**/**ml ascorbic acid**.** After 24 hours of culture, the medium was replaced with osteogenic induction medium containing various concentrations of p**-**cresol **(**10, 40, 80, and 160 µg**/**ml which equal to 94**.**22, 376**.**86, 753**.**72, and 1507**.**44 µM, respectively**)** or indoxyl sulfate **(**25, 50, 100, and 200 µg**/**ml which equal to 99**.**48, 198**.**97, 397**.**93, and 795**.**86 µM, respectively**) 26.** MSCs cultured in osteogenic induction medium without uremic toxin was used as control**.** The characteristics of osteogenic differentiated cells were evaluated at different time points by measuring osteogenic gene expression, alkaline phosphatase activity, and alizarin red S staining**.**

### Gene expression analysis

The transcriptional levels of osteogenic**-**associated genes, **(**RUNX family transcription factor 2 **(***RUNX2***)**, osteopontin **(***OPN***)**, and collagen type I** (***COL1A1***)** and senescent**-**associated genes **(**cyclin**-**dependent kinase **(**CKD**)** inhibitors *p16*, *p21*, and *p53***)** were determined**.** Briefly, cells experimentation was harvested at day 7 and 14 and completely homogenized in 1 ml Trizol **(**Invitrogen Corporation, USA**).** Then, RNA was separated using phenol**-**chloroform procedure followed by first**-**stand cDNA synthesized using cDNA synthesis kit **(**Biotechrabbit, Germany**).** For quantitative real time PCR, the PCR master mix was prepared by mixing 0**.**5 µl forward and reverse primer **(**10 µM each**)** of interested genes **(**Table 1**)**, 2 µl PCR grade water, and 5 µl KAPA SYBR Fast qPCR master mix **(**Kapabiosystems, USA**)** for each reaction and mixed with 2 µl cDNA**.** Quantitative real**-**time PCR was performed by CFX96^TM^ Real**-**Time PCR Detection System** (**Bio**-**Rad, USA**).** The difference in transcriptional levels were assessed by normalization with internal reference gene, glyceraldehyde**-**3**-**phosphate dehydrogenase **(**GAPDH**)** and presented as fold change of control**.**

### Alkaline phosphatase activity

MSCs were cultured with osteogenic induction medium in the presence or absence of indoxyl sulfate and p**-**cresol**.** After 7 and 10 days of differentiation, the cell culture medium was collected for alkaline phosphatase detection using alkaline phosphatase assay kit **(**Abcam, UK**).** Briefly, 80 µl of conditioned medium samples were incubated with 50 µl of 5 mM pNPP substrate and 10 µl of ALP enzyme solution at 25˚C for 60 minutes**.** After that, the reaction was stopped by applying 20 µl stop solution in the sample wells and standard wells**.** The colored p**-**Nitrophenol **(**pNP**)** product was measured at 405 nm using microplate reader **(**BioTek Instruments, VT, United States**).** The ALP activities **(**U**/**ml**)** were calculated according to manufacturer**'**s instructions and compared with control**.** The data were presented as relative ALP activity **(**U**/**ml**).**

### Alizarin red S staining

MSCs were cultured with osteogenic induction medium in the presence or absence of indoxyl sulfate and p**-**cresol as previously described**.** The production of bone matrix containing calcium crystals was assessed by alizarin red S staining at day 14 of differentiation**.** Cells were washed twice with PBS, fixed by incubating with 10**% (**v**/**v**)** formaldehyde at room temperature for 15 minutes, and washed twice with distill water**.** At this stage, the cells were incubated with 1 ml 40 mM alizarin red S **(**pH 4**.**1, Sigma**-**Aldrich, USA**)** at room temperature for 20 minutes with shaking**.** The excess dye was removed by washing with distill water**.** The alizarin red S staining was assessed and photographed under inverted microscopy **(**Olympus, Japan**).**

### Cell senescence analysis through senescence-associated β-galactosidase staining

To determine whether indoxyl sulfate and p**-**cresol impaired MSC osteogenesis via enhancing cellular senescence, MSCs were induced to differentiate toward osteoblast in the presence or absence of p**-**cresol **(**10, 40, 80, or 160 µg**/**ml**)** and indoxyl sulfate **(**25, 50, 100, and 200 µg**/**ml**).** After 7 days of differentiation, cells were fixed and stained with β**-**galactosidase staining solution **(**Cell Signaling Technology, USA**).** Blue color developed in senescent cells were determined under 10x objective lens of inverted microscope **(**Olympus, Japan**).** The number of positive cells were counted for the entire field and calculated by comparison with control**.** Data were presented as β**-**galactosidase positive cells relative to control**.**

### Statistics analysis

All results were performed with at least three individual experiments**.** The significant difference of mean was assessed using the paired student**'**s t**-**test or one**-**way ANOVA followed by Dunn**'**s multiple comparison test **(**GraphPad Prism software, USA**).** Data were presented as mean±standard error of the mean **(**SEM**).** P**-**value less than 0**.**05 was determined as statistical significance**.**

## Results

### Bone marrow*-*derived MSC characteristics

**Figure 1:**
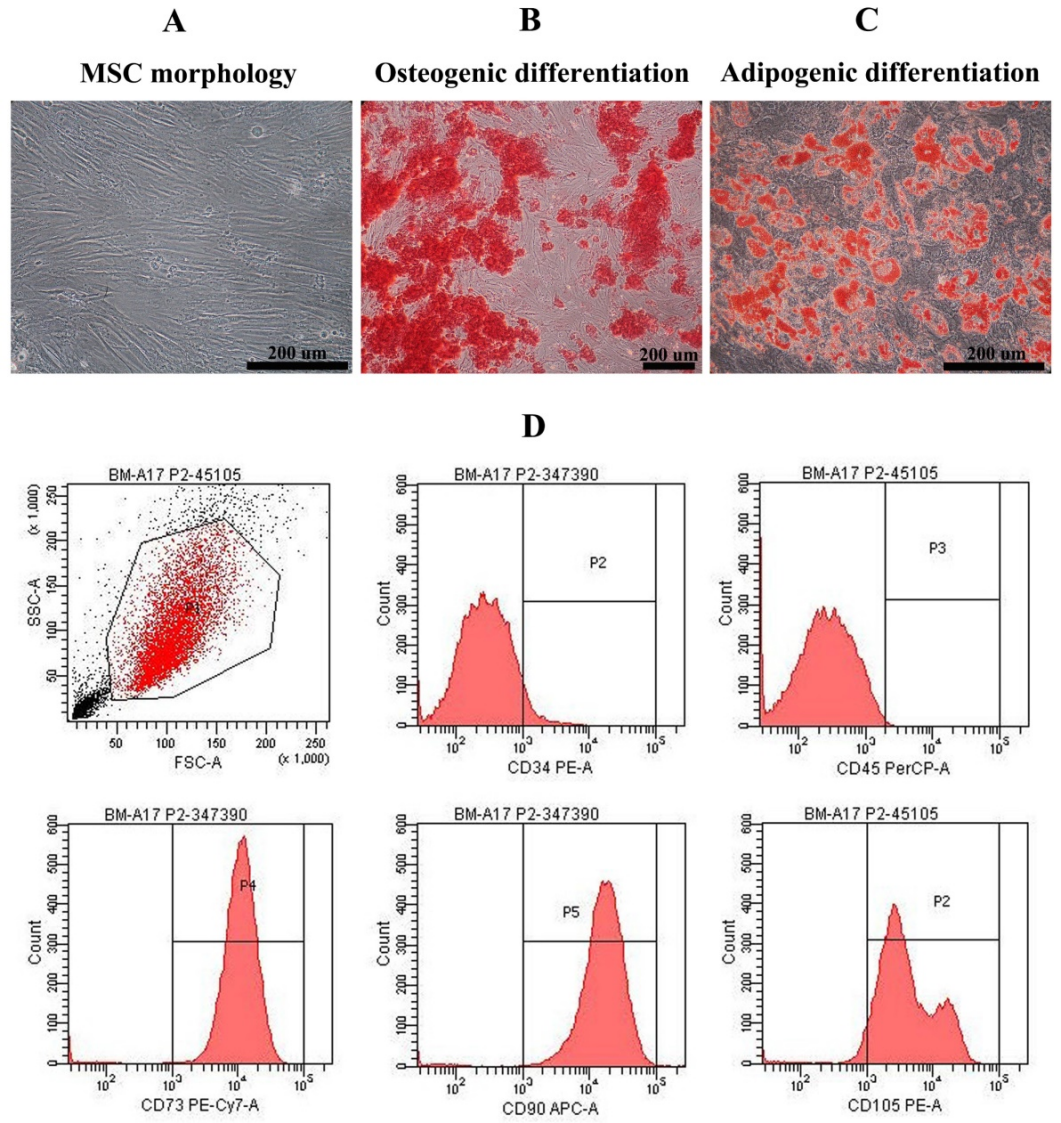
Mesenchymal stem cell characteristics: Bone marrow-derived mesenchymal stem cells exhibited fibroblast-like morphology (A) and were positive for alizarin red S staining (B) and oil red O staining (C) after cultured in osteogenic induction medium and adipogenic induction medium, respectively (Scale bar: 200 µm). Histograms demonstrated the expression of CD34, CD45, CD73, CD90, and CD105 of bone marrow-derived mesenchymal stem cell population (D).

Mesenchymal stem cells derived from bone marrow of healthy volunteers were characterized**.** They exhibited the spindle**-**shape morphology **(**Figure 1A**)** and could differentiate into osteoblast**-**, adipocyte**-**like cells showing by positive stained with alizarin red S and oil red O, respectively **(**Figure 1B, C**).** Most of bone marrow**-**derived MSCs were positive for MSC surface markers including CD105 **(**92**.**10±3**.**05**%)**, CD90 **(**98**.**80±0**.**40**%)**, and CD73** (**98**.**63±0**.**66**%)** and negative for hematopoietic markers such as CD34 **(**96**.**53±1**.**30**%)** and CD45 **(**99**.**90±0**.**10**%) (**Figure 1D**).**

### Effect of p*-*cresol and indoxyl sulfate on MSC viability

**Figure 2:**
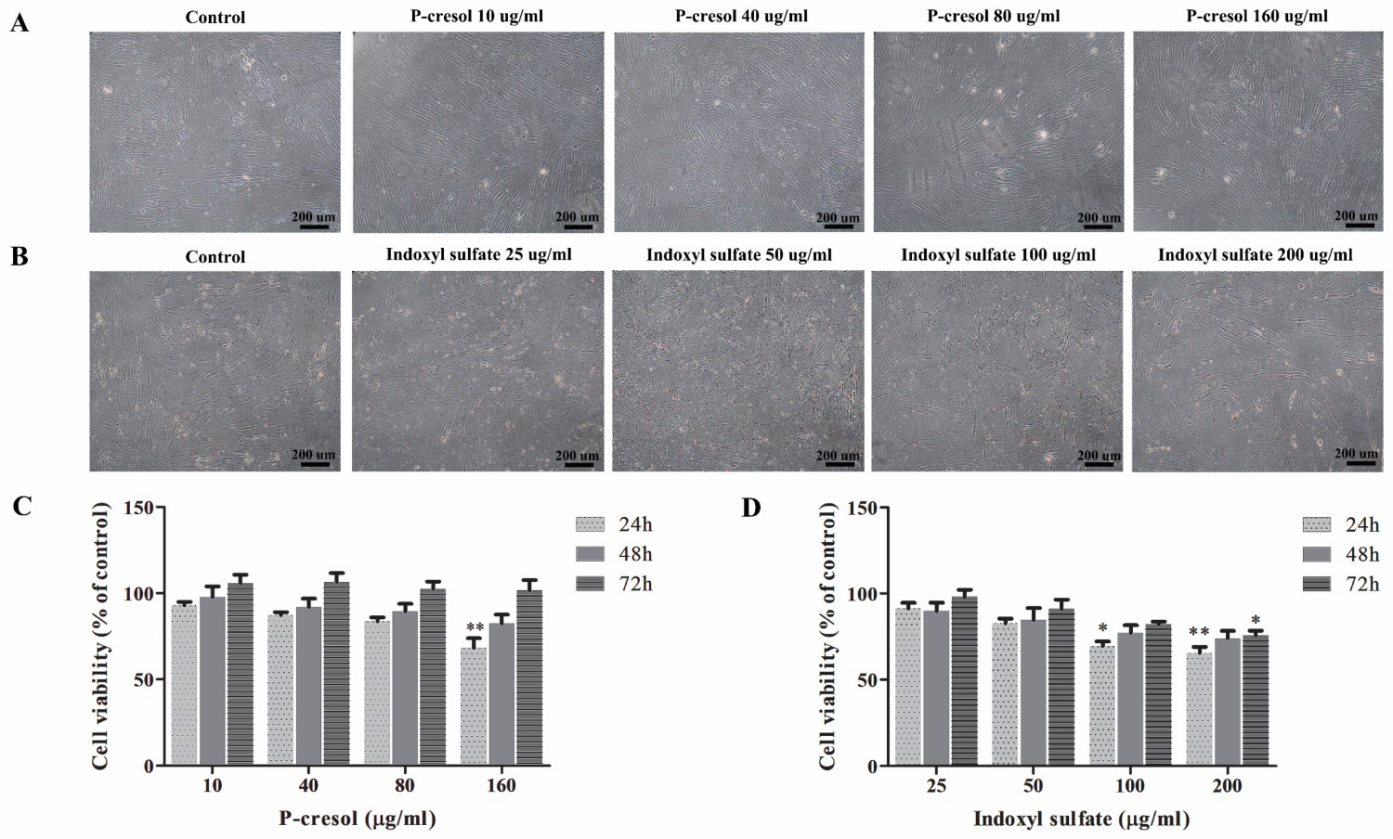
Cytotoxic effects of p-cresol and indoxyl sulfate: The morphology of MSCs was observed after incubation MSCs with various concentrations of p-cresol (A) and indoxyl sulfate (B) for 24 hours (Scale bar: 200 µm). The cell viability was determined at 24, 48, and 72 hours of incubation with p-cresol (C) and indoxyl sulfate (D) using MTT assay. The data were presented as percent viable cells of control from four individual experiments (N=4). * *P*-value < 0.05 and ** *P*-value < 0.001 as analyzed by one-way ANOVA.

To investigate the effect of uremic toxins in MSC viability, MSCs were treated with uremic toxins at various concentrations comparable to those found in CKD patients **27.** Percent cell viability was assessed after culturing either with p**-**cresol **(**10, 40, 80, and 160 µg**/**ml**) 28, 29** or indoxyl sulfate **(**25, 50, 100, and 200 µg**/**ml**) 17, 30** for 24 h, 48 h, and 72 h**.** The results demonstrated that p**-**cresol had no effect on MSC morphology compared to those cultured in control medium **(**Figure 2A**).** Meanwhile, the morphology of MSCs treated with indoxyl sulfate exhibited the shape**-**change and reduction of cell density **(**Figure 2B**).** Cell viability of MSCs treated with 160 µg**/**ml p**-**cresol for 24 hours was significantly reduced **(**68**.**15±5**.**73**%** of control**) (**Figure 2C**).** Similarly, MSC viability was declined after incubation with 100 µg**/**ml indoxyl sulfate **(**69**.**48±2**.**71**%** of control**)** and 200 µg**/**ml indoxyl sulfate **(**65**.**51±3**.**59**%** of control**)** for 24 hours**.** In addition, MSC treated with 200 µg**/**ml indoxyl sulfate for 72 hours revealed significantly reduction of MSC viability to 75**.**78±2**.**72**%** of control **(**Figure 2D**).**

### P*-*cresol impaired osteogenic differentiation potential of MSCs

**Figure 3:**
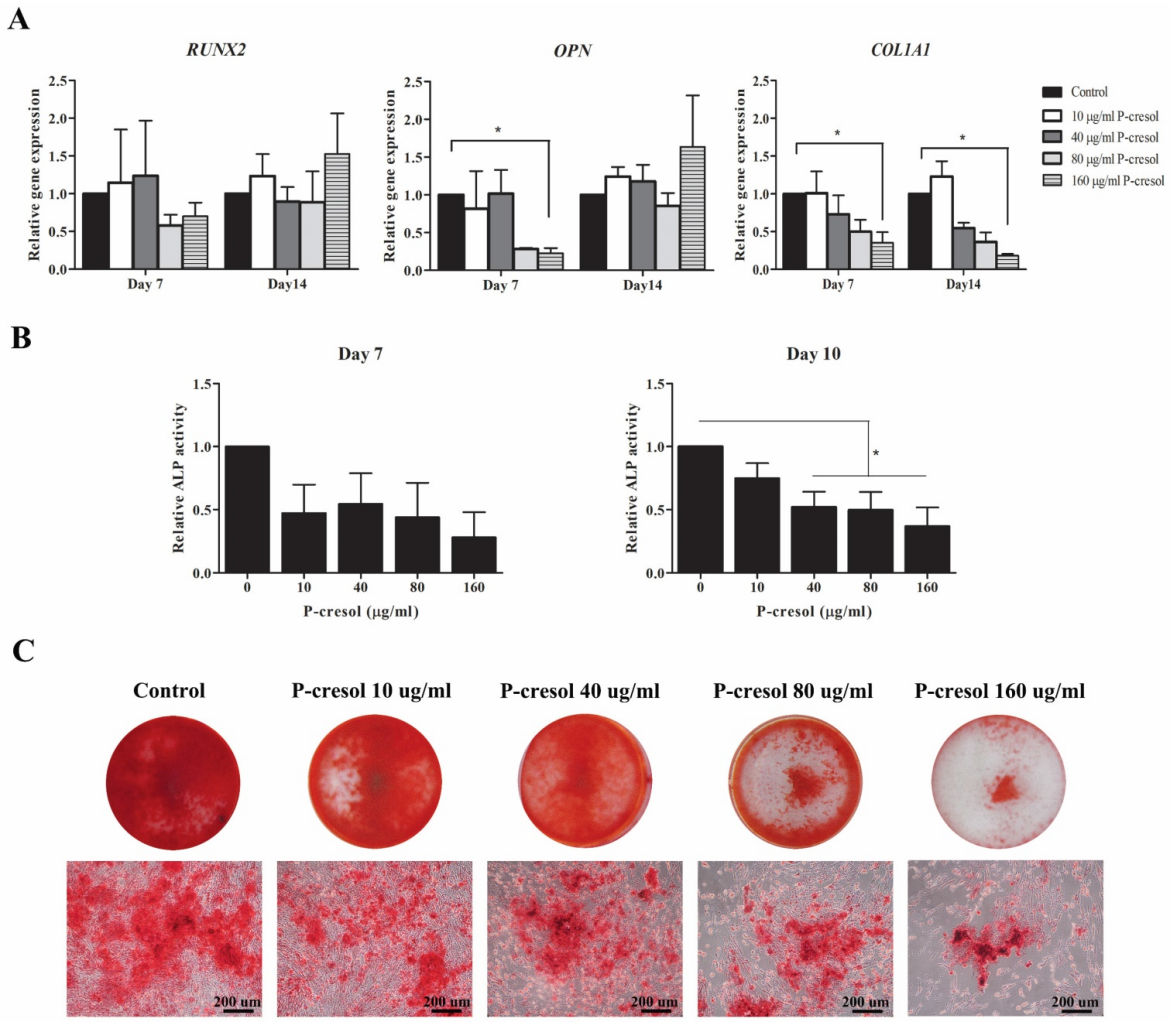
The effects of p-cresol on osteogenesis of MSC: Osteogenic differentiation of MSCs were determined which included the expression of osteogenic-associated genes, ALP activity, and mineralization. The expression levels of osteogenic-associated genes; *RUNX2*, *OPN*, and *COL1A1* were examined after treatment with p-cresol for 7 days and 14 days of osteogenic differentiation (A). The alkaline phosphatase (ALP) activity was assessed after treatment with p-cresol (10, 40, 80, and 160 µg/ml) for 7 day and 10 day of differentiation (B). The data were presented as relative ALP activity (U/ml) from four individual experiment (N=4). * *P*-value < 0.05 comparison with control. Alizarin red S staining was investigated for calcium deposition at day 14 of differentiation (C) (Scale bar: 200 µm).

To examine the effects of p**-**cresol on MSC**-**osteoblast differentiation, MSCs were cultured in osteogenic induction medium supplemented with various concentrations of uremic toxins for 14 days**.** The transcriptional levels of osteogenic**-**associated genes including *RUNX2, OPN,* and *COL1A1* were investigated at day 7 and day 14 of differentiation**.** As shown in Figure 3A, the significant different decreased of *OPN* gene expression was observed in MSCs treated with 160 µg**/**ml p**-**cresol at day 7 of differentiation as well as *COL1A1* at day 7 and 14**.** The decline of ALP activity was observed in MSC**-**treated with all concentrations of p**-**cresol at day 7 and day 10 **(**Figure 3B**)** and revealed significantly reduced in MSCs treated with 40 µg**/**ml p**-**cresol **(**0**.**52±0**.**12 U**/**ml**)**, 80 µg**/**ml p**-**cresol **(**0**.**50±0**.**14 U**/**ml**)**, and 160 µg**/**ml p**-**cresol **(**0**.**37±0**.**15 U**/**ml**)** for 10 days**.** The result of alizarin red S staining further confirmed the adverse effects of p**-**cresol on osteogenic differentiation capacity of MSCs**.** The exposure with p**-**cresol during *in vitro* osteogenesis revealed the dose**-**dependently inhibitory effect in mineralization, represented by alizarin red S staining **(**Figure 3C**).** Calcium deposition of MSC**-**osteoblast was dramatically depleted in 80**-**160 µg**/**ml p**-**cresol treatment**.** The results indicating p**-**cresol accumulation impaired osteogenic differentiation properties of MSCs**.**

### Indoxyl sulfate influenced osteogenic differentiation potential of MSCs

**Figure 4:**
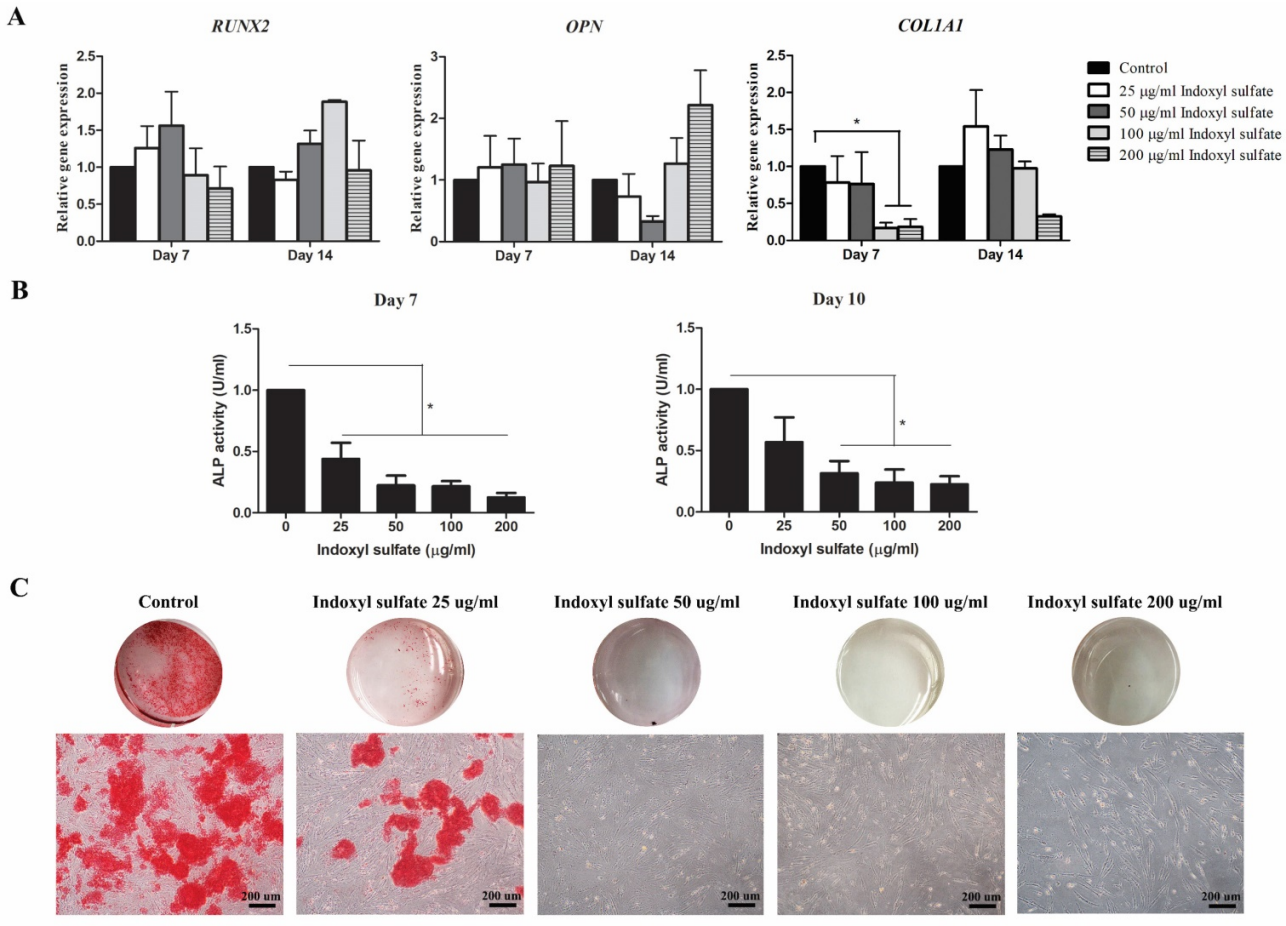
Effects of indoxyl sulfate (IS) on osteogenic differentiation potential of MSCs: Bar graph demonstrate the expression levels of osteogenic-associated genes; *RUNX2, OPN*, and *COL1A1* at day 7 and 14 of differentiation after treatment with indoxyl sulfate relative to control (A). Alkaline phosphatase activity assessed after treatment with indoxyl sulfate (25, 50, 100, and 200 µg/ml) for 7 day and 10 day of differentiation (B). The data were presented as relative ALP activity (U/ml) from four individual experiment (N=4). * *P* value < 0.05 comparison with control. Alizarin red S staining was assessed for calcium deposition at day 14 of differentiation (C) (Scale bar: 200 µm).

The effect of indoxyl sulfate **(**IS**)** in osteogenic differentiation was also evaluated**.** The expression of osteogenic**-**associated genes *RUNX2, OPN,* and* COL1A1* were investigated and only the reduction of *COL1A1* transcripts was observed at day 7 and 14 of MSCs treated with 100**-**200 µg**/**ml indoxyl sulfate** (**Figure 4A**).** The relative ALP activities of MSCs showed dose dependently reduced in MSCs treated with indoxyl sulfate at day 7 and 10 **(**Figure 4B**).** Furthermore, mineralization of osteoblastic differentiation was illustrated by alizarin red S staining**.** The result showed the low alizarin red S stained in MSCs with 25 µg**/**ml indoxyl sulfate treatment **(**Figure 4C**).** Moreover, the negative staining was observed in the conditions that MSCs were treated with 50**-**200 µg**/**ml indoxyl sulfate during osteogenic differentiation**.** Thus, it is clearly evidence that indoxyl sulfate inhibits MSC**-**osteoblastic differentiation**.**

### P*-*cresol and indoxyl sulfate induced MSC senescence during osteogenic differentiation

**Figure 5:**
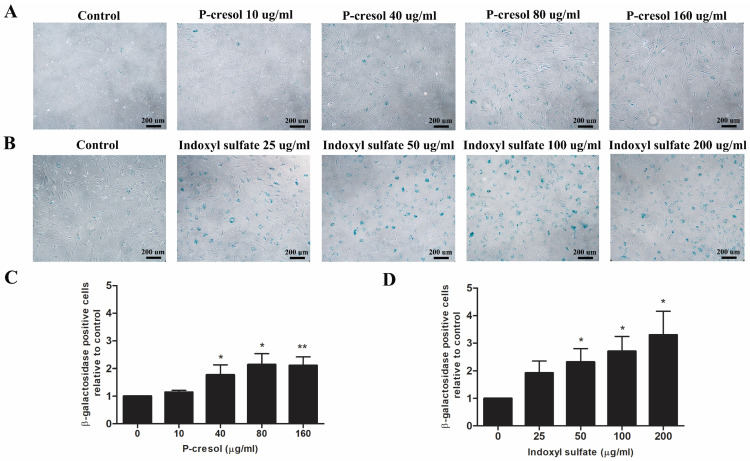
The role of p-cresol and indoxyl sulfate on MSC senescence during osteogenic differentiation: Senescence-associated betagalactosidase staining demonstrated the blue color developed in senescent cells after exposure to p-cresol (A) and indoxyl sulfate (B) on day 7 of osteogenic differentiation (Scale bar: 200 µm). The number of β-galactosidase positive cells were counted and presented as β-galactosidase positive cells relative to control (C-D). * *P*-value < 0.05 and ** *P*-value < 0.01 as compared with control.

To determine whether uremic toxins influence MSC**-**osteoblast differentiation through the cellular senescence, the treated cells were stained with senescence**-**associated beta**-**galactosidase **(**SA**-**β**-**gal**)** after 7 days of differentiation**.** The SA**-**β**-**gal positive cells were significantly increased in condition with 40 µg**/**ml p**-**cresol **(**1**.**77±0**.**36 folds of control**)**, 80 µg**/**ml p**-**cresol **(**2**.**11±0**.**31 folds of control**)**, and 160 µg**/**ml p**-**cresol **(**2**.**11±0**.**31 folds of control**) (**Figure 5A, C**).** For indoxyl sulfate, the number of MSCs positive for SA**-**β**-**gal was significantly increase in 50 µg**/**ml **(**2**.**31±0**.**49 folds of control**)**, 100 µg**/**ml **(**2**.**71±0**.**54 folds of control**)**, and 200 µg**/**ml **(**3**.**30±0**.**86 folds of control**)** indoxyl sulfate treatment **(**Figure 5B, D**).**

### P*-*cresol upregulated p21 expression during osteogenic differentiation

**Figure 6:**
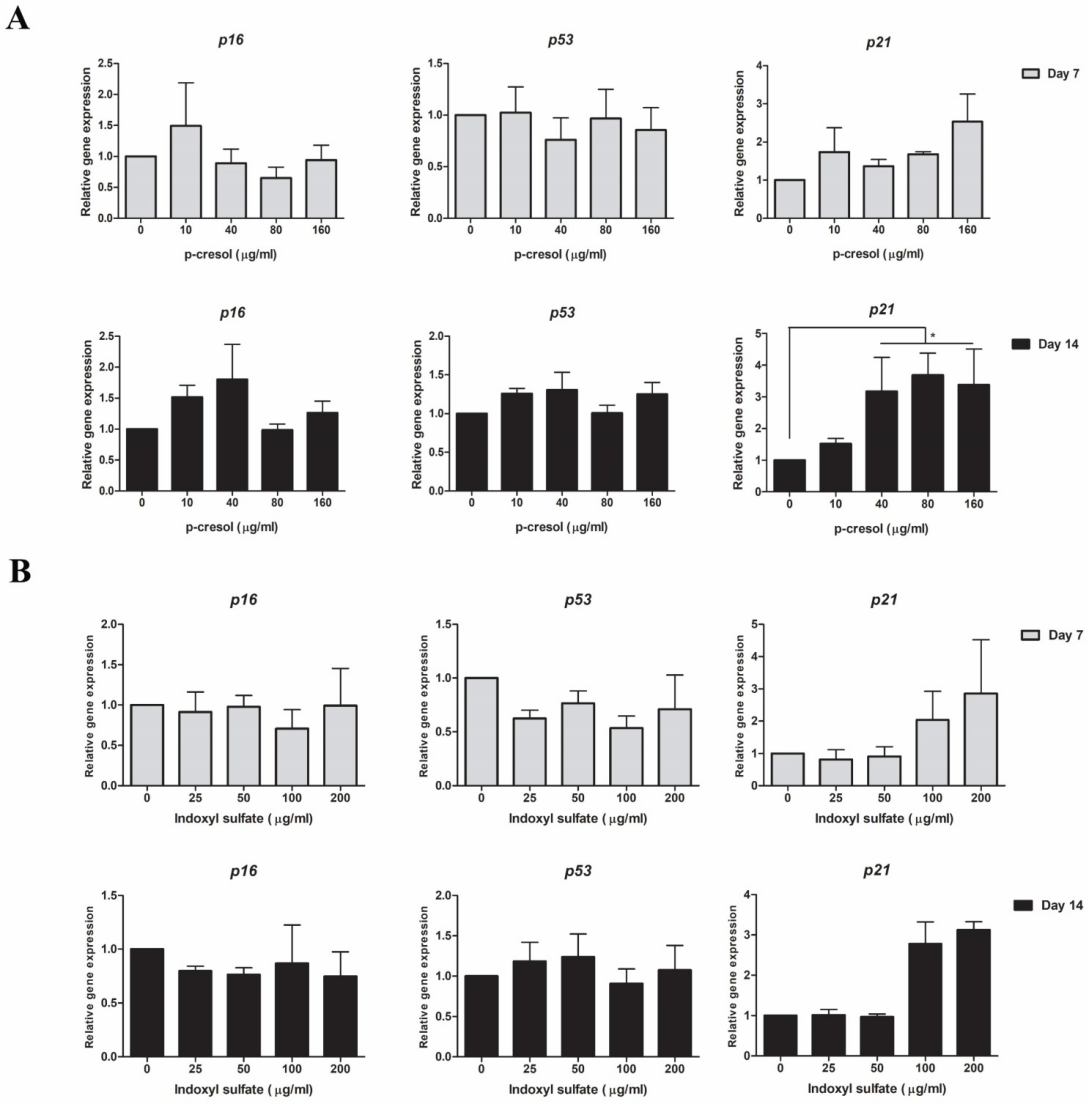
The transcriptional levels of senescence-associated genes after p-cresol and indoxyl sulfate treatment: The expression of senescence-associated genes including *p16*, *p53*, and *p21* was investigated after exposure to p-cresol (A) and indoxyl sulfate (B) at day 7 and day 14 of osteogenic differentiation. * *P*-value < 0.05 as compared with control.

The transcriptional levels of key senescence**-**associated genes including *p16*, *p53*, and *p21* were determined in MSCs treated with either p**-**cresol or indoxyl sulfate at day 7 and day 14 of osteogenic differentiation**.** At day 7, only *p21* transcriptional levels was gradually increased in cells with uremic toxins treatment** (**Figure 6A**).** At day 14 of differentiation, the expression of *p21* was significantly upregulated in 40 µg**/**ml **(**3**.**18±1**.**07 folds of control**)**, 80 µg**/**ml **(**3**.**69±0**.**69 folds of control**)**, and 160 µg**/**ml **(**3**.**38±1**.**13 folds of control**)** p**-**cresol treatment**.** However, there were no significantly different in *p53* and *p16* expression investigated**.** For indoxyl sulfate treatment, the transcriptional levels of *p21* were upregulated in 100 µg**/**ml **(**2**.**78±0**.**54 folds of control**)** and 200 µg**/**ml **(**3**.**12±0**.**21 folds of control**)** indoxyl sulfate treatment at day 7 and 14 of differentiation** (**Figure 6B**).** The expression of *p53* and *p16* were not significantly different among indoxyl sulfate treatment and untreated cells**.** The result suggesting that during the MSCs**-**osteoblastic differentiation, either p**-**cresol or indoxyl sulfate affected the expression of a senescent**-**associated gene, *p21***.**

## Discussion

Protein**-**bound uremic toxins have been considered as one of the major issues inducing CKD progression **31, 32.** Accumulation of uremic toxins in CKD patients not only accelerated the progression of diseases but also contributed to the occurrence of various complications including cardiovascular disease, anemia, and bone abnormalities**.** The bone defect is one of the most common complications found in CKD patients**33.** Imbalance of bone formation and bone resorption is associated with several factors such as an abnormal mineral level, a decrease of vitamin D, and a disruption of parathyroid hormone **(**PTH**)** signaling **21.** Previous study showed that indoxyl sulfate attenuated osteoclast differentiation and impaired osteoclast functions**20.** Thus, high levels of protein**-**bound uremic toxins in CKD patients may associate with bone abnormalities and impair the balance between bone formation and bone resorption**34.** To better understand the role of uremic toxins on osteogenesis, this study used MSCs as a model for studying osteogenesis**.** During osteogenic differentiation, MSCs were treated with uremic concentrations of p**-**cresol or indoxyl sulfate**.** Subsequently, the sequence of osteogenic differentiation and functionalities of MSC**-**osteoblastic cells including osteogenic transcriptional expression, alkaline phosphatase activity analysis, and mineralization assay were investigated at different periods**.**

The cytotoxic effects of p**-**cresol and indoxyl sulfate were determined by culture MSCs with a certain uremic toxin for 24, 48, and 72 hours**.** In agreement with previous study, p**-**cresol treatment did not alter MSC morphology, however MSCs survival was significantly reduced when incubation with high concentration of p**-**cresol** (**160 µg**/**ml**) 26.** Moreover, our study demonstrated that the MSC viability of the p**-**cresol treated cells could be recovered as same as of the control after long term culture**.** Thus, it is possible that the remaining cells may obtain high proliferation capacity to compensate the cytotoxic effects of uremic toxins **35.** In addition, the alteration of MSCs morphology was observed in MSCs cultured with indoxyl sulfate even at low concentration**.** Together with the finding of lower cell viability in indoxyl sulfate treated MSCs, this suggested that protein**-**bound uremic toxins** (**IS**)** could exert strong cytotoxic effects on MSCs**.** Previous studies showed that indoxyl sulfate could decrease MSC survival by increasing the production of reactive oxygen species and oxidative stress**36.** In addition, indoxyl sulfate also modulated the activity of other cell types such as endothelial cells and astrocytes which may lead to an increase risk for developing cardiovascular disease and neurological complication in CKD patient**16, 37.** Our current study found the indoxyl sulfate and p**-**cresol contain cytotoxic effects in MSCs survival with the dissimilar effect**.** Thus, it is possible that the cytotoxic effects of p**-**cresol and indoxyl sulfate may mediate via different mechanisms or else each uremic toxin could affect the osteogenesis through each stages of bone development which finally lead to bone formation defect**.**

This study clearly demonstrated that uremic toxins, p**-**cresol and indoxyl sulfate impaired osteogenesis and osteoblast functions**.** We demonstrated that indoxyl sulfate can affect cell growth in contrary with p**-**cresol**.** However, both p**-**cresol and indoxyl sulfate downregulated the expression of key osteogenic**-**associated gene; *COL1A1* which encoded for collagen type I protein**.** Collagen type I is the major bone matrix which influenced the early stage of osteoblast differentiation**23.** Interestingly, neither p**-**cresol nor indoxyl sulfate affected *RUNX2* transcripts**.** Our result clearly support the previous study which demonstrated the decreases of *COL1A1* levels but not *RUNX2* levels in MSCs cultured in uremic milieu**35.** Thus, these protein**-**bound uremic toxins may differently modulate osteogenesis and impact via other mechanisms apart from directly regulation of *RUNX2* transcripts and reduction of cell growth**.** Kim et al suggested that indoxyl sulfate could inhibit osteoblast cell line differentiation through organic anion transport** (**OAT**)** which mediate the uptake of indoxyl sulfate **22.** Furthermore, p**-**cresol and indoxyl sulfate had an impact on alkaline phosphatase **(**ALP**)** activity, an important enzyme required for mineralization of bone matrix**.** Our results showed that p**-**cresol and indoxyl sulfate gradually decreased alkaline phosphatase **(**ALP**)** activity in MSC**-**osteoblastic cells in dose**-**dependent manner which lead to the impairment of mineralization accounting for the osteoblastic cell functionality**.** In addition, the alizarin red S staining showed that both p**-**cresol and indoxyl sulfate dose**-**dependently reduced calcium deposition of MSC**-**osteoblastic cells**.** This finding supported the previous study which suggested that p**-**cresyl sulfate could reduce proliferation capacity of osteoblast**38.** Role of indoxyl sulfate was shown to dramatically inhibited MSC**-**osteoblastic cell mineralization and induced osteoblast cell line apoptosis which finally attenuated its maturation**22.** Taken together, the high concentration of both uremic toxins influenced the maturation of bone demonstrated by *in vitro* study of MSCs**-**osteoblast differentiation**.**

The underlying mechanisms of those protein**-**bound uremic toxins on osteoblastogenesis are poorly characterized**.** Our results clearly demonstrated that p**-**cresol and indoxyl sulfate markedly induced MSC**-**osteoblastic cells to undergo senescence during osteogenic differentiation**.** Growing evidences indicating the cellular senescence could diminish multipotent differentiation potential of MSCs**39.** Meanwhile, the upregulation of *p21* which play a critical role in inducing cell cycle arrest was observed in both p**-**cresol and indoxyl sulfate treatment**.**

The expression of *p16* was not much different among MSCs treated with uremic toxin which in contrast with the osteoblast isolated from bone tissue of old mice**40.** This imply that osteoblastic senescent cells among vary pathogenic conditions could be influenced by the different stimuli that need to be determined**.** The former study demonstrated the impact of indoxyl sulfate on inducing mesenchymal stem cell senescence through the increase of *Bax* and *p53* expression **22.** Although p21 is a downstream signaling of p53, the alteration of mRNA level of *p53* did not investigated in the uremic toxin treated cells in our study**.** It is possible that uremic toxins might affect p53 and phosphorylation p53 expression at translational levels resulting in p21 upregulation**.** In addition, growing evidences have demonstrated the regulation of p21 expression through p53 independent manner**41-44.** Previous studies demonstrated the function of p21 in a p53**-**deficient cancer cell line was associated with cell growth arrest and cellular senescence**41, 43.** In addition to p53, p21 level could be controlled by multiple factors at both post**-**transcriptional and post**-**translational regulation **42.** Another factor such as insulin**-**like growth factor**-**binding protein**-**related protein 1 **(**IGFBP**-**rP1**)** was identified involving with cellular senescence**.** Transfection of IGFBP**-**rPr1 in breast cancer cell line upregulated p21 expression and increased senescence**-**associated galactosidase **(**SA**-**β**-**gal**)** positive cells **44.** Taken together, the p**-**cresol and indoxyl sulfate could partially induce MSC**-**osteoblastic senescence by the activation of p21 via p53**-**independent pathway resulting in the regulation of cell cycle progression**.** Currently, the bone problems in patient with kidney diseases was caused by bone aging which influenced by osteoblast senescence**45.** The underlying mechanism could be explored in part from this study, suggested by the increasing of mesenchymal**-**osteoblast aging induced by exposure of cell with high concentration of p**-**cresol and indoxyl sulfate**.**

Collectively, the results from our study illustrated that protein**-**bound uremic toxins, p**-**cresol and indoxyl sulfate exert deleterious impacts on osteoblastogenesis and osteoblast functions *in vitro***.** These effects are at least mediated partially through the activation of p21 lead to the induction of MSC**-**osteoblast senescence by p**-**cresol and indoxyl sulfate**.** Accumulation of protein**-**bound uremic toxins may be the primary cause of bone dysfunction in CKD patients**.** Therefore, levels of protein**-**bound uremic toxins found in CKD patients, should be critically concerned**.** Reducing protein**-**bound uremic toxin levels will protect osteoprogenitor cells from senescence**.** Moreover, improvement of therapeutic approach to eliminate senescent cells will reduce cellular aging and alleviate bone loss in CKD patients**.**

## Figures and Tables

**Table 1:** The sequences of primers for quantitative RT-PCR.

Gene name	Forward primer sequences	Reverse primer sequences
*RUNX2*	5**'-**AACCCAGAAGGCACAGACAG**-**3**'**	5**'-**GCCTGGGGTCTGTAATCT GA**-**3**'**
*OPN*	5**'-**ACAGCCAGGACTCCATTGAC**-**3**'**	5**'-**GGGGACAACTGGAGTGAAAA**-**3**'**
*COL1A1*	5**'-**GGGCAAGACAGTGATTGAATACA**-**3**'**	5**'-**GGATGGAGGGAGTTTACAGGAA**-**3**'**
*P16*	5**'-**TGAGGGTTTTCGTGGTTCAC**-**3**'**	5**'-**TGGTCTTCTAGGAAGCGGC**-**3**'**
*P21*	5**'-**GATGAGTTGGGAGGAGGCAG**-**3**'**	5**'-**CTGAGAGTCTCCAGGTCCAC**-**3**'**
*P53*	5**'-**ATGATTTGATGCTGTCCCCG**-**3**'**	5**'-**CAAGAAGCCCAGACGGAAAC**-**3**'**
*GAPDH*	5**'-**CAACTACATGGTTTACATGTTCCAA**-**3**'**	5**'-**CAGCCTTCTCCATGGTGGT**-**3**'**
